# Central nervous system tumours in children in Ibadan, Nigeria: a histopathologic study

**DOI:** 10.11604/pamj.2016.24.34.9344

**Published:** 2016-05-09

**Authors:** Gabriel Olabiyi Ogun, Amos Olufemi Adeleye, Taiwo Olabimpe Babatunde, Olufunmilola Abimbola Ogun, Ayodeji Salami, Biobele Jotham Brown, Effiong Akang

**Affiliations:** 1 Department of Pathology, College of Medicine, University of Ibadan, and University College Hospital, Ibadan, Nigeria; 2 Division of Neurological Surgery, Department of Surgery, College of Medicine, University of Ibadan and University College Hospital, Ibadan, Nigeria; 3 Neuro-Ophthalmology Unit, Department of Ophthalmology, College of Medicine, University of Ibadan, and University College Hospital, Ibadan, Nigeria; 4 Oncology Unit, Department of Paediatrics, College of Medicine, University of Ibadan and University College Hospital, Ibadan, Nigeria

**Keywords:** CNS neoplasms, paediatric, children, Ibadan, Nigeria

## Abstract

**Introduction:**

Contrary to some earlier teachings that central nervous system (CNS) tumours are uncommon in black children, these neoplasms are the fourth most common paediatric tumours in Ibadan. Our centre is the major referral centre for CNS tumours in Nigeria. The last major study of paediatric CNS neoplasms from Ibadan was in 1985. An update of the data on paediatric CNS neoplasms at our centre is presented.

**Methods:**

A retrospective review of all histologically diagnosed CNS tumours in children (0-14 years) from January 2001 to December 2010 from the database of the Department of Pathology, University College Hospital, Ibadan, Nigeria was done. The cases were classified using the 2007 WHO Classification of Tumours of the Central Nervous System and were also based on their supratentorial and infratentorial locations.

**Results:**

Seventy-seven tumours, 44 in males, were included in the study. Astrocytic tumour comprised 20 cases, embryonal tumours 15, ependymal tumours 15, germ cell tumours 6, sellar tumours (all craniopharyngiomas) 9 and other histological types- 12 cases. Thirty-seven were WHO Grade 1, eleven Grade 2, ten Grade 3 and nineteen Grade 4 neoplasms. Thirty-six cases were supratentorial and thirty-eight were infratentorial in location. The most common tumours in this series were pilocytic astrocytomas, medulloblastomas, craniopharyngiomas and ependymomas in that order.

**Conclusion:**

Childhood CNS tumours are being increasingly diagnosed in our centre. This is largely explained by the recent expansion of the available neurosurgical services.

## Introduction

Only three decades ago, it was widely believed that the incidence of gliomas was relatively low in black children, when compared with Caucasian children [1, 2]. Although central nervous system(CNS) neoplasms are the second most common paediatric tumours in Caucasians series [3–5], they only rank as the fourth most common paediatric tumour in the past studies in Ibadan, Nigeria [1, 2]. Furthermore, a recent review of paediatric neoplasms from Ibadan documented a six-fold increase, from 2.2% to 12.9%, in the relative frequency of paediatric CNS neoplasms over a period of five decades [6]. Another study in 2007 revealed that 37% of all CNS neoplasms at our centre occurred in children [7]. It is speculated that a similar trend can be inferred for other centres in Nigeria. The reason that can be allude for this is the recent modest increase in access to high-resolution diagnostic tools such as computed tomography (CT) scanning and magnetic resonance imaging (MRI). Our nation has also recently begun to witness a progressive increase in the critical mass of competent clinical neuroscientists including those in neurosurgery, adult and paediatric neurology, neuroradiology, neuropathology and neuro-ophthalmology, as well as some other ancillary medical staff. The CNS is an extremely complex and specialised system known to harbour an array of approximately 130 primary neoplasms [8]. The pattern of CNS tumours in childhood differs in terms of the histopathologic subtypes as well as the anatomical location from those in adults. Generally, the main tumour groups in children are astrocytomas (38-50%), ependymoma (8-14%), primitive neuroectodermal tumours (PNET), including medulloblastoma(16-25%), and other gliomas (4-16%) [9]. The distinction between benign and malignant neoplasms is blurred in the brain; in that all CNS neoplasms may eventually be attended by fatal raised intracranial pressure (ICP), irrespective of the degree of histological differentiation. In this respect, and especially so in many countries in sub-saharan African, many childhood CNS neoplasms often only come to clinical awareness after their initial non-specific symptomatology has evolved to more terminal ones like those of raised ICP. There is a gross lack of data on the demographic patterns of paediatric CNS tumours in many developing countries of the world due to non availability of reliable data collection, poor infrastructure for cancer registries and many other logistic constraints. Since the last major study of childhood CNS neoplasm from our centre in 1985, there has been no further attempt at a review targeted primarily at childhood CNS tumours in Ibadan, Nigeria, the nation's flagship centre of clinical and academic neuroscience [10]. The aim of this present study is therefore to update the data on childhood CNS tumours at our centre, describe the histopathological pattern, and determine the anatomical distribution of the tumours. We also made an attempt at comparison with the previous two studies from our centre as well with a few studies from other parts of the world.

## Methods

This is a retrospective data base study of childhood tumours of the central nervous system (CNS) diagnosed in the Department of Pathology, University College Hospital, Ibadan, Nigeria between January 2001 and December 2010. All CNS neoplasms in children aged 0-14 years were retrieved from the files and records of the department including autopsy records and from the records of Ibadan Cancer Registry (our department falls under its jurisdiction). Also a systematic review of the literature was also performed. Cases that were not histopathologically confirmed were excluded from the study. The data abstracted from patients records include age (stratified into 0-4 years, 5-9 and 10-14), gender, intracranial location of the tumour (either supra or infratentorial), and the specific histological diagnosis. The cases were classified using the 2007 WHO Classification of Tumours of the Central Nervous System after reconfirmation of the diagnosis [8]. Immunohistochemistry was used in the confirmation of some cases where necessary. The data obtained was entered into the Statistical Programme for Social Sciences, version 20 (SPSS Inc, IL, USA) and subjected to analysis. This study was conducted in compliance with the guidelines of the Helsinki declaration on biomedical research in human subjects. Confidentiality of the identity of the patients and personal health information was maintained.

## Results

Seventy seven tumours were included in the study out of which 44 (57%) were males with a male to female ratio of 1.3:1. The ages of the patients ranged from 29 days of life to 14 years with a mean of 7.2 years and a median of 7 years. Thirty-one cases (40.3%) occurred in the age bracket 0-4 years, 27 (35.1%) in 5-9 years and 19 cases (24.7%) in 10-14 years. In all there were 36 cases (46.8%) that were WHO Grade 1, 12 cases (15.6%) were Grade 2, 10 cases (13.0%)- Grade 3, and 19 (24.7%) were Grade 4 neoplasms (Table 1). In other words, 37.7% of the childhood tumours of the CNS in Ibadan were of the histologically malignant variety. Majority, 16 out of 29 (55.1%) of these occurred in the 0-4 years age group (Table 1). Overall, astrocytic tumours comprised 20 (25.9%), embryonal tumours 15 (19.5%), ependymal tumours 15 (19.5%), sellar tumours- all were craniopharyngiomas 9 (11.7%), germ cell tumours 6 (7.8%), meningeal tumours 7 (9.0%) and other histological types made up the remaining 5 cases.

**Table 1:** Age group distribution and WHO grades of CNS neoplasms of children in Ibadan

	WHO GRADE			
Age group (yrs)	I	II	III	IV
**0-4**	11	4	6	10
**5-9**	15	4	3	5
**10-14**	10	4	1	4
**TOTAL**	36	12	10	19

**Astrocytic neoplasms**: The 20 cases recorded comprised of 17 grade one tumours (16 pilocytic astrocytomas and one case of subependymal giant cell astrocytoma), a single Grade 2 tumour (fibrillary astrocytoma) and two glioblastomas.

**Embryonal neoplasms**: These comprised 13 medulloblastomas, and a case each of ganglioneuroblastoma and ependymoblastoma.

**Ependymal tumours**: These comprised a single case of myxopapillary ependymoma, 10 ependymomas and 4 cases of anaplastic ependymoma. Table 2 show details for all the tumours in this series. Thirty-eight (50.6%) cases were infratentorial and thirty seven (49.4%) were supratentorial in location. There were two spinal region neoplasms. Table 3 shows the details of the distribution of the tumours.

**Table 2:** Paediatric CNS neoplasms in Ibadan, Nigeria: gender and age group distribution of all tumours in the series

Morphological subtype	GENDER			AGE GROUPSl		
	M	F	M:F	0-4	5-9	10-14
**ASTROCYTIC TUMOURS**						
Pilocytic astrocytoma	11	5	2.2:1	5	9	2
Fibrillary astrocytoma	1	-		-	1	-
Subependymal giant cell astrocytoma	-	1		-	-	1
Glioblastoma	1	1	1:1	1	1	-
**EPENDYMAL TUMOUR**						
Myxopapillary ependymoma	-	1		-	1	-
Ependymoma	7	3	2.3:1	3	3	4
Anaplastic ependymoma	3	1	3:1	2	2	-
**EMBRYONAL TUMOURS**						
Medulloblastoma	5	8	1:1.6	8	3	2
Ganglioneuroblastoma	1	-		-	1	-
Ependymoblastoma	1	-		1	-	-
**NEURONAL AND MIXED NEURONAL- GLIAL TUMOURS**						
Ganglioglioma	-	1		-	1	-
Dysembryoplastic neuroepithelial tumour	1	-		-	-	1
**PINEAL REGION TUMOURS**						
Pinealoblastoma	1	-		-	-	1
**TUMOURS OF CRANIAL & PARASPINAL NERVES**						
Neurofibroma*	-	1		-	-	1
**TUMOURS OF THE MENINGES**						
Meningioma	2	3	1:1.5	2	1	2
Haemangiopericytoma	-	1		1	-	-
Lipoblastoma*	1	-		1	-	-
**GERM CELL TUMOURS**						
Yolk sac tumour	2	2	1:1	3	-	1
Immature teratoma	-	1		1	-	-
Mixed germ cell tumour	1	-		-	1	-
**TUMOURS OF SELLAR REGION**						
Craniopharyngioma	5	4	1.3:1	3	3	3
**METASTATIC TUMOURS**						
Metastatic neuroblastoma	1	-		-	-	1
**TOTAL**	44	33		31	27	19

* One case each of these group of tumours occured in the spinal canal

**Table 3:** Paediatric CNS neoplasms in Ibadan, Nigeria: morphologic sub-types in relation to anatomical locations

Morphological sub-types	Infratentorial	Supratentorial
Astrocytic tumours	16	4
Ependymal tumours	6	9
Embryonal tumours	14	1
Neuronal and mixed-glial tumours	1	1
Pineal region tumours	0	1
Germ cell tumours	1	5
Sellar region tumours	0	9
Meningeal tumours^*^	0	6
Nerve sheath tumours^*^	0	0
Metastatic tumour	0	1
**TOTAL**	**38**	**37**

^+^One case each in these groups of tumours occurs in the spinal canal. They both were not included in this table

## Discussion

A recent 5-year (2004-2008) review of data from the Ibadan cancer registry shows that, overall, CNS tumours accounted for 2.5% of all newly diagnosed registered tumours [11]. However, childhood tumours were not well segregated from those of adults in the publication. This lack of segregation is what is commonly observed in studies on CNS tumours from developing countries [7, 12]. In many developing countries, including Nigeria, there are significant logistic constraints to a complete hospital data collection and population based studies are virtually impossible to carry out. Hence, most times, hospital based studies are the only source of useful data available to describe the pattern of paediatric CNS tumours. These, in spite of their known unreliability are then sometimes extrapolated for the population [10, 12]. This current study is the second attempt at detailed documentation of childhood CNS tumours from our centre. The first attempt covered a period of 22 years (1960-1982) [10] and was a report dating back 3 decades. A second study that covered an 11 year period was an overview of CNS tumours in adults and children with some separation in both categories [12]. Table 4 shows a comparison of these two earlier studies with the current one. Consistently, astrocytic tumours have remained the commonest primary CNS neoplasms in children at our hospital though with a downward trend in the percentage from 39% to 25.9% in the current study [10, 12]. A distinctive finding in the current study is the almost complete absence of secondary tumours. A comparison to the two previous studies from our centre by Aghadiuno et al and Olasode et al show a high percentage of secondary involvement of the brain by Burkitt's lymphoma [10, 12]. None was observed in the present study. This trend of sustained decreasing incidence of Burkitt′s lymphoma as an entity and with its attendant secondary involvement of the CNS has been observed in the last two decades [6]. We are of the opinion that the significant improvement in the treatment of malaria and some measure of improved nutrition in children from our country have led to this trend. Furthermore, ependymomas which were of insignificant proportion in the previous two studies have emerged to become the second commonest tumours after the astrocytic tumours. Because of the corresponding relative decline in astrocytic neoplasms observed in the present study, it may be that some neoplasms recorded as astrocytic tumours in the previous two studies might actually have been ependymal neoplasms.

**Table 4:** Paediatric CNS neoplasms in Ibadan, Nigeria: comparison of the two previous studies from Ibadan with the current study

	aAghadiuno et al ^10^ 1960-1982 89 cases	bOlasode et al^12^ 1980-1990 75 cases	Current Study 2001-2010 77 cases
**ASTROCYTIC TUMOURS**	**35 (39%)**	**31 (41.3%)**	**20 (25.9%)**
Glioblastoma	-	1(1.3%)	2(2.6%)
**EPENDYMAL TUMOURS**	**4 (4%)**	**-**	**15(19.5%)**
**EMBRYONAL TUMOURS**	**12 (13%)**	**8(10.6%)**	**15(19.5%)**
Medulloblastoma	12(13%)	8(10.6%)	13(16.8%)
**NEURONAL AND MIXED NEURONAL- GLIAL TUMOURS**	-	-	**2( 2.6%)**
**OLIGODENDROGLIAL TUMOURS**	**2 (2%)**	**4(5.3%)**	**-**
**PINEAL REGION TUMOURS**	-	-	**1(1.3%)**
Pinealoblastoma	-		
**TUMOURS OF CRANIAL & PARASPINAL NERVES**	-	-	**1(1.3%)**
**TUMOURS OF THE MENINGES**	**1(1%)**	**-**	**7( 9%)**
Meningioma	1	-	5
**GERM CELL TUMOUR**	**2(2%)**	**1(1.3%)**	**6(7.8%)**
Immature teratoma	2	1	1(1.3%)
**TUMOURS OF SELLAR REGION**	**9(10%)**	**15 (20%)**	**9( 11.7%)**
Craniopharyngioma	8(9%)	12(16%)	9
Pituitary adenoma	1(1%)	3	-
**METASTATIC TUMOURS**	**21(23%)**	**13(17.3%)**	**1(1.3%)**
Neuroblastoma	11(12%)	NS	1
Burkitt's lymphoma	9(10%)	7(9.3%)	-
Myeloid leukaemia	1(1%)	NS	-

a,b Vascular tumours as a tumour group were classified in these series. Three cases in each series was not included in the table NS- Not Stated

A comparison of the current study with some other studies from different parts of the world, (Table 5), shows that generally astrocytic tumours are the commonest neoplasms in most regions of the world. Though, astrocytic tumours account for 25.9% in our series, their frequency is much higher ranging from 30.5% to 47.3% in series from Europe, Asia and South America [13–20]. In this study ependymoma was the second ranked tumour, accounting for 19.5% of cases. This is in contrast to all the other studies we have compared with the current study, in which the frequency ranged from 4.8% to 10.5% [14, 16, 17, 21]. The retrospective nature of this analysis precludes any meaningful speculations to explain this trend. Medulloblastomas accounted for 16.8% of cases in our study and ranked the third most common tumour. This ranking and percentage of its relative frequency is comparable to that of 10 to 18.4% recorded in studies from Asia and Europe as illustrated in Table 5 [13–18]. The exception to this was a study from Pakistan where medulloblastoma accounted for 45.6% of cases in that series [21].

**Table 5:** Paediatric CNS neoplasms in Ibadan, Nigeria: comparison of current study findings with some other series from different parts of the world

Study	Country	Total Number of cases	Maximum Age (years)	Relative frequency of four commonest CNS tumours				M:F ratio for all tumours	Mean age (years)	Supra- tentorial (%)	Infra-tentorial (%)
				1	2	3	4				
Peris-Bonet et al.^13^	Europe	19,531+	<15	Astrocytic tumours (40%)	PNET (including medulloblastoma)	Ependymoma	NS	NS	NS	NS	NS
Kaatsch et al.^14^	Germany	3,268^a^+	<15	Astrocytic tumours (41.7%)	Medulloblastoma (18.1%)	Ependymoma (10%)	PNET (6.4%)	1.2:1	10.7	46.2+	48.3+
Zhou D et al.^15^	China	1,485	<17	Astrocytic tumours (30.5%)	Craniopharyngioma (18.5%)	Medulloblastoma (14.6%)	Germ cell tumours (7.8%)	1.6:1	NS	61.9	38.1
Asirvatham et al.^16^	India	1,043	<18	Astrocytic tumours (47.3%)	Medulloblastoma (11.4%)	Craniopharyngioma (9.7%)	Ependymoma (4.8%)	1.7:1	10.9	53.3	40.6
Mehrazin et al.^17^	Iran	619	<15	Astrocytic tumours (40.4%)	Medulloblastoma (18.4%)	Ependymoma (10.5%)	Craniopharyngioma (8.8%)	1.4:1	8.8	51	49
*Makino et al.^18^	Japan	210	<15	Astrocytic tumours (35.7%)	Germ cell tumours (14.3%)	Craniopharyngioma (10.5%)	Medulloblastoma (10.0%)	1.3:1	NS	NS	NS
Ahmed et al.^21^	Pakistan	81	<15	PNET (49.4%)++	Astrocytic tumours (34.6%)	Ependymoma (10%)	Mixed glioneuronal tumours (5%)	2.5:1	8.8	33.3	66.7
Current study	Nigeria	77	<15	Astrocytic tumours (25.9%)	Ependymoma (19.4%)	Medulloblastoma (16.8%)	Craniopharyngioma (11.6%)	1.3:1	7.2	49.4	50.6

a Intracranial and intraspinal germ cell tumours were excluded

* These were population based studies

** included Medulloblastoma which accounted for 45.6**%** of tumours in the series NS- Not stated

+ Spinal Cord and meningeal tumours not included

Generally boys are usually more affected than girls with regards to paediatric CNS tumour. The ratio of 1.3:1 is similar to studies from Germany, Iran and Japan [14, 17, 18]. A higher male:female ratio was recorded in studies from China and India [15, 16]. Furthermore a 2.5:1 male preponderant ratio was recorded in the Pakistani study, though gender bias favouring boys having easy accesses to health care as a socio-cultural practice might be responsible [12, 16]. The mean age in this series was 7.3 years. This is somewhat relatively lower when compared to studies with similar maximum age limit of 14 years inclusion criteria. The studies from Germany, Iran, Pakistan had mean age of 10.7, 8.8 and 8.8 years respectively [14, 17, 21]. This difference may not be explainable from the age structure in our country where the higher percentage of the population is children. Higher mean age is observed in studies with higher maximum age limit as inclusion criteria for such studies. In the current study, there was almost an equal ratio between supra- and infra- tentorial tumours. This is similar to the finding of Kaatsch et al and Mehrazin et al from Germany and Iran respectively. However, studies from China, South Korea, India and Brazil show a preponderance of supratentorial tumours [4, 15, 16, 20, 22]. Infratentorial tumours were more commonly seen in the studies from Pakistan [21, 23].

## Conclusion

In conclusion astrocytic tumours, ependymomas, medulloblastoma and craniopharyngiomas account for about 73% of intracranial tumours of children aged 0-14 years of age in this study. Certain differences exist in the frequency of occurrence of the tumours between this study and others. However, this represents a picture of the paediatric brain tumours that came to our attention and that were histologically diagnosed in the particularly challenging African environment where we practice.

### What is known about this topic

- Central nervous system tumour are rare in Black children;
- Astrocytic tumours are the commonest CNS tumours in children.

### What this study adds

- Increasing diagnostic capabilities at UCH has resulted in an increased identification of CNS tumors;
- This is a good audit of the UCH experience in terms of histopathologic pattern of CNS tumours.

## References

[1] Williams AO (1975). Tumors of childhood in Ibadan, Nigeria. Cancer.

[2] Olisa EG, Chandra R, Jackson MA, Kennedy J, Williams AO (1975). Malignant tumors in American black and Nigerian children: a comparative study. J Natl Cancer Inst.

[3] Akang EEU (2000). Epidemiology of cancer in Ibadan: tumours in childhood. Arch Ib Med..

[4] Rosemberg S, Fujiwara D (2005). Epidemiology of pediatric tumors of the nervous system according to the WHO 2000 classification: a report of 1,195 cases from a single institution. Childs Nerv Syst ChNS Off J Int Soc Pediatr Neurosurg.

[5] Scotting PJ, Walker DA, Perilongo G (2005). Childhood solid tumours: a developmental disorder. Nat Rev Cancer.

[6] Ojesina AI, Akang EEU, Ojemakinde KO (2002). Decline in the frequency of Burkitt's lymphoma relative to other childhood malignancies in Ibadan, Nigeria. Ann Trop Paediatr.

[7] Idowu O, Akang EEU, Malomo A (2007). Symptomatic primary intracranial neoplasms in Nigeria, West Africa. J Neurol Sci Turk..

[8] Louis DN, Ohgaki H, Wiestler OD, Cavenee WK, Burger PC, Jouvet A (2007). The 2007 WHO classification of tumours of the central nervous system. Acta Neuropathol (Berl)..

[9] Parkin D, Kramarova E, Draper G, Masuyer E, Michaelis J, Neglia J (1998). International incidence of childhood cancer.

[10] Aghadiuno PU, Adeloye A, Olumide AA, Nottidge VA (1985). Intracranial neoplasms in children in Ibadan, Nigeria. Childs Nerv Syst..

[11] Ogunbiyi J, Fabowale A, Ladipo A (2010). Cancer incidence and Top ten cancers in eleven local governments in Ibadan and its environs, 2004-2008: Ibadan Cancer Registry, Nigeria. Technical report.

[12] Olasode BJ, Shokunbi MT, Aghadiuno PU (2000). Intracranial neoplasms in Ibadan, Nigeria. East Afr Med J.

[13] Peris-Bonet R, Martínez-García C, Lacour B, Petrovich S, Giner-Ripoll B, Navajas A (2006). Childhood central nervous system tumours--incidence and survival in Europe (1978-1997): report from Automated Childhood Cancer Information System project. Eur J Cancer.

[14] Kaatsch P, Rickert CH, Kühl J, Schüz J, Michaelis J (2001). Population-based epidemiologic data on brain tumors in German children. Cancer.

[15] Zhou D, Zhang Y, Liu H, Luo S, Luo L, Dai K (2008). Epidemiology of nervous system tumors in children: a survey of 1,485 cases in Beijing Tiantan Hospital from 2001 to 2005. Pediatr Neurosurg..

[16] Asirvatham JR, Deepti AN, Chyne R, Prasad MSN, Chacko AG, Rajshekhar V (2011). Pediatric tumors of the central nervous system: a retrospective study of 1,043 cases from a tertiary care center in South India. Childs Nerv Syst.

[17] Mehrazin M, Yavari P (2007). Morphological pattern and frequency of intracranial tumors in children. Childs Nerv Syst.

[18] Makino K, Nakamura H, Yano S, Kuratsu J-I, Kumamoto Brain Tumor Group (2010). Population-based epidemiological study of primary intracranial tumors in childhood. Childs Nerv Syst.

[19] Jain A, Sharma MC, Suri V, Kale SS, Mahapatra AK, Tatke M (2011). Spectrum of pediatric brain tumors in India: a multi-institutional study. Neurol India.

[20] Pinho RS, Andreoni S, Silva NS, Cappellano AM, Masruha MR, Cavalheiro S (2011). Pediatric central nervous system tumors: a single-center experience from 1989 to 2009. J Pediatr Hematol Oncol.

[21] Ahmed N, Bhurgri Y, Sadiq S, Shakoor KA (2007). Pediatric brain tumours at a tertiary care hospital in Karachi. Asian Pac J Cancer Prev.

[22] Cho K-T, Wang K-C, Kim S-K, Shin S-H, Chi JG, Cho B-K (2002). Pediatric brain tumors: statistics of SNUH, Korea (1959-2000). Childs Nerv Syst.

[23] Nasir S, Jamila B, Khaleeq S (2010). A retrospective study of primary brain tumors in children under 14 years of age at PIMS, Islamabad. Asian Pac J Cancer Prev..

